# Rheological Studies of PMMA–PVC Based Polymer Blend Electrolytes with LiTFSI as Doping Salt

**DOI:** 10.1371/journal.pone.0102815

**Published:** 2014-07-22

**Authors:** Chiam–Wen Liew, R. Durairaj, S. Ramesh

**Affiliations:** 1 Centre for Ionics University of Malaya, Department of Physics, Faculty of Science, University of Malaya, Kuala Lumpur, Malaysia; 2 Department of Mechanical and Material Engineering, Faculty of Engineering & Science, Universiti Tunku Abdul Rahman, Setapak, Kuala Lumpur, Malaysia; University of Nebraska-Lincoln, United States of America

## Abstract

In this research, two systems are studied. In the first system, the ratio of poly (methyl methacrylate) (PMMA) and poly (vinyl chloride) (PVC) is varied, whereas in the second system, the composition of PMMA–PVC polymer blends is varied with dopant salt, lithium bis (trifluoromethanesulfonyl) imide (LiTFSI) with a fixed ratio of 70 wt% of PMMA to 30 wt% of PVC. Oscillation tests such as amplitude sweep and frequency sweep are discussed in order to study the viscoelastic properties of samples. Elastic properties are much higher than viscous properties within the range in the amplitude sweep and oscillatory shear sweep studies. The crossover of 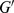 and 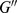 is absent. Linear viscoelastic (LVE) range was further determined in order to perform the frequency sweep. However, the absence of viscous behavior in the frequency sweep indicates the solid-like characteristic within the frequency regime. The viscosity of all samples is found to decrease as shear rate increases.

## Introduction

Solid polymer electrolyte (SPE) is an attractive candidate in the area of electrical power generation and storage systems. It is a great substitution for conventional liquid electrolyte because it has potential to solve the shortcomings of liquid electrolytes such as corrosive solvent leakage and harmful gas. In addition, it possesses high electrochemical and thermal stabilities as well as low volatility [1]–[2]. Other features are light in weight, easy to process due to its flexibility, high energy density, high ionic conductivity and high automation process [3]–[5]. Wide range of applications is another advantage for SPE, ranging from small scale production of commercial secondary lithium batteries to advanced high energy electrochemical devices, such as chemical sensors, electrochromic windows, fuel cells, supercapacitors, analog memory devices, dye–sensitizers solar cells (DSSCs), hybrid electrical vehicle (EV) and start–light–ignition (SLI) which serves as traction power source for electricity [6]–[7].

As usual, ionic conductivity is the main aspect in the polymer electrolytes. However, the ionic conductivity is closely related to the viscosity of a sample. For instance, low viscosity of sample will produce higher ionic conductivity by forming more voids within the polymer matrix. Therefore, the physical properties of samples are well studied in order to understand the effect of physical characteristics onto bulk ionic transportation further. In general, all materials can behave as solid or liquid. Solid–like materials flow as liquids under a sustained shear stress at very long time (or equivalently at very low frequency). It can therefore be concluded that all materials are viscoelastic and it strongly depends on the timescale of deformation. Rheology study is a well known physical technique to study the flow and deformation properties of materials [8]. It is composed of two main tests: rotational test and oscillation test. Rotational test is used to determine to the flow behavior of sample when the stress is applied onto the sample, whereas the viscoelastic behavior is explored by oscillation test. Examples of rotational test are flow curve, viscosity curve and thixotropy which defined as structure recovery. Oscillation tests include amplitude sweep (AS), frequency sweep (FS) and temperature sweep (TS). Amplitude sweep is probed to study the linear visco–elastic (LVE) range of the sample, whereas frequency sweep is the oscillatory equivalent to the rotational flow curve which scrutinizes the long–term structural stability of samples. Ahmad et al. found out that the storage modulus increases when PVC is added to polystyrene (PS) [9].

There are several approaches have been done to increase the ionic conductivity of polymer electrolytes. The crystalline polymer–based electrolytes showed low ionic conductivity due to high degree of crystallinity. The mobile ions migrate slowly in the crystalline phase. Therefore, amorphous polymer, PMMA is used in this present work. However, PMMA–based polymer electrolytes are very brittle and exhibit low mechanical integrity. Therefore, PVC is blended with PMMA as stiffener to improve the mechanical strength of polymer electrolytes [10]. PMMA–PVC polymer blend electrolytes have been widely studied in last decade including our published papers [5], [7], [10]–[11]. Up to date, there are not much rheology studies on the PMMA–PVC polymer blend electrolytes, except our own published work [11]. The effect of adding ionic liquids and nano–sized fillers onto the rheological properties of PMMA–PVC polymer blend electrolytes were investigated in our previous work. However, this present work shows different objective. We will examine the effect different concentration of PVC and LiTFSI onto the rheological properties of PMMA–PVC polymer blend electrolytes in this work.

## Experimental

### Materials

In this study, PMMA with an average molecular weight of 350000 gmol^−1^ (Aldrich), high molecular weight of PVC (Fluka), inorganic dopant salt LiTFSI (Fluka) and solvent tetrahydrofuran (THF) (J.T. Baker) were used without further purification.

### Sample preparation

Prior to the preparation of the polymer electrolytes, LiTFSI was dried at 100°C for 1 hour to eliminate trace amounts of water in the material. Appropriate amounts of PMMA, PVC and LiTFSI were dissolved in THF. The weight ratio is expressed as weight percentage. Two polymeric systems are employed in this study. For the first system, the prepared compositions were [*x*PMMA–(1–*x*)PVC], where *x* is 0.1, 0.5 and 0.7 and the weight ratio of polymer blend to lithium salt is maintained at 90∶10, as tabulated in Table 1. In contrast, Table 2 shows the variation of mass fraction of polymer blend and lithium salt with a fixed ratio of 70 wt% PMMA to 30 wt% PVC for second system. The solution was continuously stirred for 24 hours to obtain a homogenous mixture at room temperature and subjected to rheometer.

**Table 1:** Weight compositions of PMMA, PVC and LiTFSI for first polymer electrolyte system and their designations.

Designation	Composition of PMMA: PVC: LiTFSI				
	PMMA		PVC		LiTFSI
	Weight (g)	Weight percentage (wt%)	Weight (g)	Weight percentage (wt%)	Weight (g)
PMMA	1 g	100	-	-	-
PMMA–PVC	0.70	70	0.30	30	-
PE 3	0.63	70	0.27	30	0.10
PE 5	0.45	50	0.45	50	0.10
PE 9	0.09	10	0.81	90	0.10

**Table 2:** Weight compositions of PMMA, PVC and LiTFSI for second polymer electrolyte system and their designations.

Designation	Composition of PMMA: PVC: LiTFSI					
	PMMA		PVC		LiTFSI	
	Weight (g)	Weight percentage (wt%)	Weight (g)	Weight percentage (wt%)	Weight (g)	Weight percentage (wt%)
SPE 3	0.595	59.5	0.255	25.5	0.15	15
SPE 6	0.490	49.0	0.210	21.0	0.30	30
SPE 8	0.420	42.0	0.180	18.0	0.40	40

### Characterizations

Rheological measurements were performed by means of Anton–Paar Physica MCR 301 rheometer. The geometry was a cone plate with a diameter of 60 mm and gap height of 0.056 mm. All the rheological measurements were carried out at ambient temperature with a fresh sample each time. The parameters were in logarithm scale. Amplitude sweep studies were examined in a log strain ramp from 0.001% to 150% with 5 points per decade at angular frequency of 10 s^−1^. For time setting, 30 measuring points with 5 s duration had been set in the whole measurement. On the other hand, all the data acquisitions for frequency sweep tests were carried out over a frequency range between 0.01 s^−1^ and 0.1 s^−1^ with 20 measuring points per 5 s duration. A fixed shear rate parameter, ranging from 0.1 s^−1^ to 100 s^−1^, with 32 measuring points per 5 s duration was recorded as the profile of viscosity curves. The sample was sandwiched between stainless steel cone plate geometry and a stationary bottom plate. The excessive sample was trimmed thereafter. Solvent trap was used to slow down the evaporation process of solvent in the sample. Before starting the test, about 1 min of rest period was allowed, in order to stabilize the normal forces between the polymer electrolytes and the cone plate.

Amplitude sweep is designed to measure the visco–elastic properties of samples without the disruption of the internal structures [12]. A sinusoidal stress as a function of the angular frequency (*ω*) and the stress amplitude (*τ_o_*) is applied on the samples [13]. Both the applied stress and resultant strain are expressed as below: 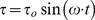
(1)

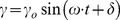
(2)where *ω* is angular frequency and *ω* = 2π*f*, *f* being the frequency in Hertz (Hz); *t* is the time and *δ* is phase shift. The ratio of the applied shear stress to the maximum strain is known as complex modulus (G^*^). It is a measure of the resistance of a material to deform, as expressed below:
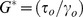
(3)


The complex modulus can be divided into two main components: elastic and viscous properties. The proportion of elastic (solid) properties is determined by storage modulus (*G′*) which is defined as a measure of elastic energy. On the contrary, loss modulus (*G″*) or dissipation of viscous energy, is employed to characterize viscous (liquid) behavior. The complex modulus is expressed as functions of storage and loss modulus:
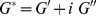
(4)


The elastic and viscous properties represent strain in–phase and out–of–phase, respectively [12]. Both storage and loss moduli are defined as:
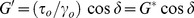
(5)

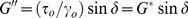
(6)where *τ_o_* is stress amplitude; *γ_o_* is strain amplitude and *δ* is the phase angle between the stress and strain (or known as loss angle) within 0<θ<90°. In principle, the material is pure elastic system when the resultant stress wave is exactly in phase with the strain, along with *δ* = 0. On the other hand, when the rate of change of sinusoidal oscillation is at a maximum and the strain is becoming to zero, the perfect viscous sample is described if the resultant stress wave is exactly π/2 (or equivalent as 90°) out of phase with the imposed deformation [12]–[13].

## Results and Discussion

### Amplitude sweep

#### 1) First system

**Figure 1:**
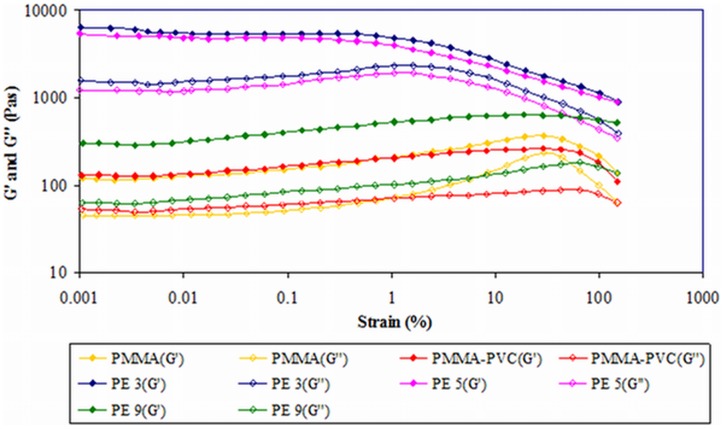
Amplitude sweep of first system.

In this system, the variation of composition ratio of PMMA to PVC is employed with 10 wt% of LiTFSI as a manipulated variable. Figure 1 depicts the storage (*G′*) and loss (*G″*) moduli as a function of imposed deformation. As shown in this figure, *G′* values are much greater than *G″* values over the whole strain range and at all concentrations, implying that all the samples behave as solid. No crossover point of *G′* and *G″* (or more commonly known as yield point or flow point) is observed for all the samples within the strain range. This observed result indicates that the transition of solid behavior to liquid behavior is absent with increasing strain of the samples [14]. General trends of all samples are observed. An almost constant range is observed and defined as linear visco–elastic (LVE) range. In general, LVE range is the range of stability of a sample before it undergoes the structural changes. The longer the range, more stable the sample is. As shown in Figure 1, PE 3 shows a wider stability range than PE 5. This discloses that PE 3 is more stable than PE 5. It still remains highly elastic even though longer elongation is produced. Beyond this constant range, for pure PMMA, PMMA–PVC and PE 9, both of *G′* and *G″* show an increment. At high strain (around 30%), i.e. when the sample is more deformed, pure PMMA illustrates a sudden plateau region in *G′* and *G″*. It is then followed by a decrease. The brief jump in *G″* is evocative of enhancement of liquid properties at large change in dimensions by deformation. On the contrary, PMMA–PVC and PE 9 have same patterns of plot. The deformation of all the samples has been proven by showing the decreased *G′* and *G″* values at high strain. It divulges that the transient coordination bonds have been destroyed which in turn contributes to the structural change in the samples and therefore reduces *G′* and *G″* values.

**Figure 2:**
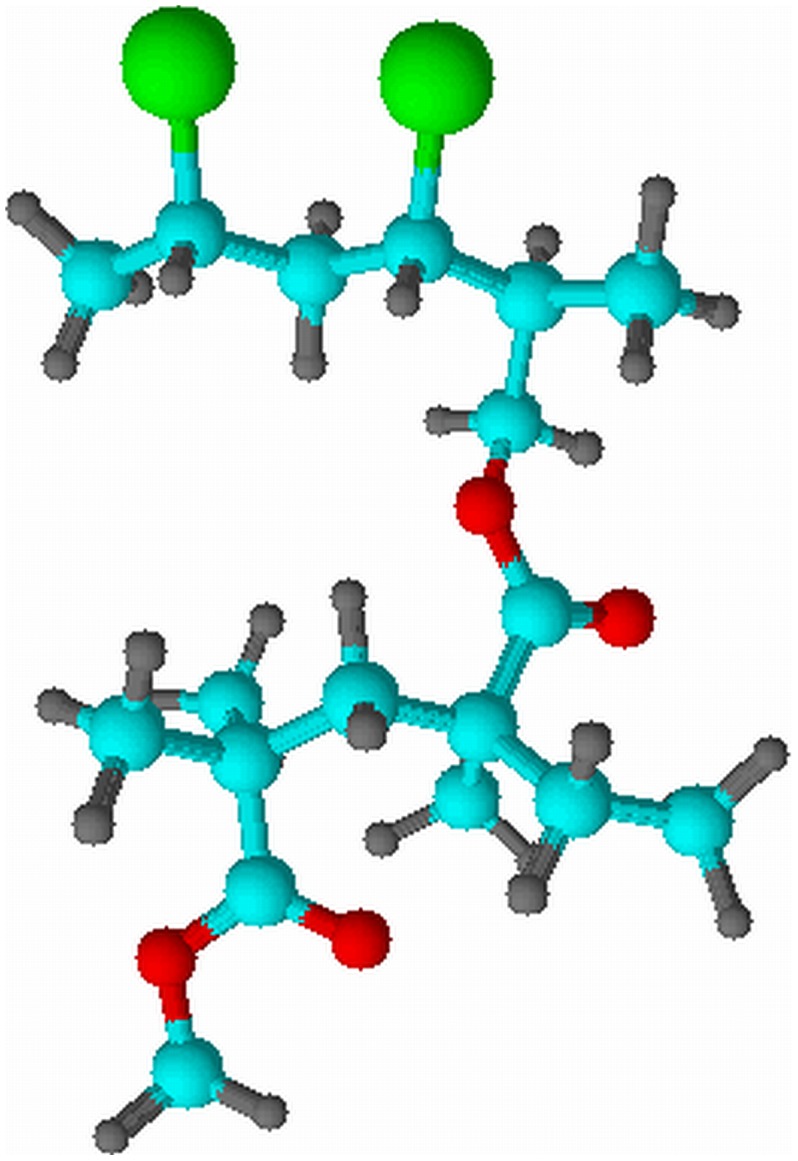
The cross–linkage between PMMA and PVC.

Pure PMMA shows the lowest values of *G′* and *G″* at rest state. However, both of values are increased when PVC is introduced into the PMMA sample, inferring more solid–like behavior of PMMA–PVC polymer blends system. It is suggestive of formation of transient cross–linking between PMMA and PVC macromolecules, as shown in Figure 2. The dehydrochlorination process promotes the cross–linkage by forming the chemical bonding of methoxyl group (O–CH_3_) from PMMA with PVC polymer backbone [7]. Consequently, the PVC polymer chains resist the viscous flow behavior [9]. At zero–strain, PE 3 and PE 5 illustrate abrupt jump of *G′* and *G″* of about two orders of magnitude, whereas a gradual increase is observed for PE 9. This signifies the higher solid characteristic and mechanical properties of samples. The phenomenon arises from the cross–linking effect between PMMA and PVC, as explained above. Another perspective is complexation between LiTFSI and polymer blends through hydrogen bonding. PE 5 has similar trend as PE 3, but it manifests lower *G′* and *G″* than PE 3. It is indicative of heterogeneous distribution of PMMA and PVC blends with increasing PVC loadings [9]. This is mainly attributed to the agglomeration of similar phase of PVC and thus ultimately leads to formation of phase separation in the polymer matrix. Similar explanation is also applied onto PE 9 as it demonstrates lower *G′* and *G″* values in comparison to PE 3 and PE 5. At high PVC content, PVC molecules tend to aggregate and thus interrupt the intermolecular bonding between PMMA and PVC, favoring the phase separation. Eventually, it reduces the solid characteristic of sample.

#### 2) Second system

**Figure 3:**
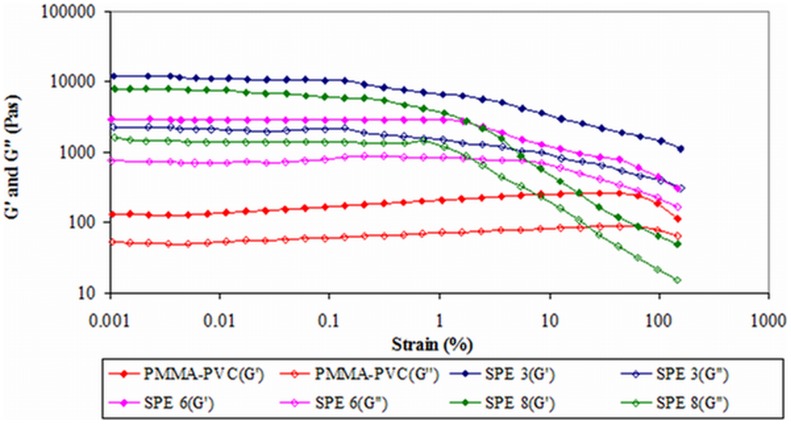
Amplitude sweep of second system.

**Figure 4:**
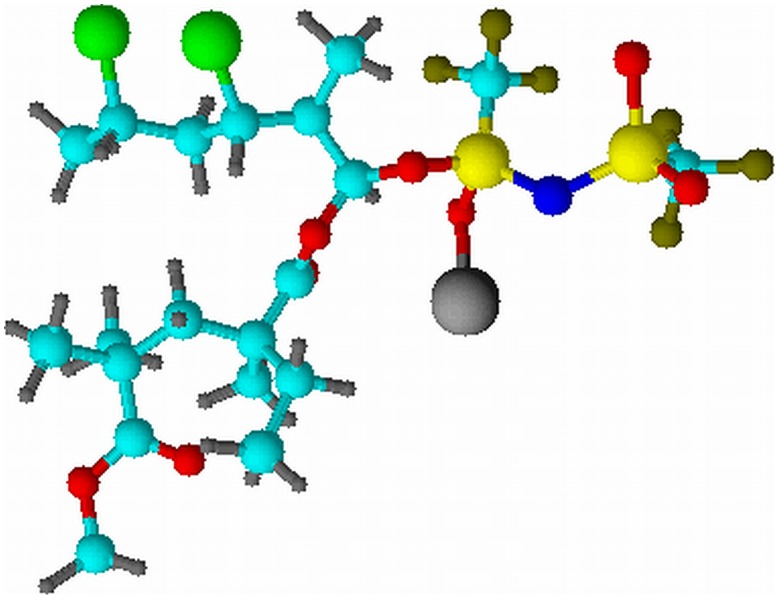
Hydrogen bonding between LiTFSI and polymer blends.

The effect of LiTFSI dopant salt is investigated in this study. Figure 3 shows the amplitude sweep of this polymer system. According to the figure, the yield point is absent within the strain regime. This implies that there is no behavioral transition in this polymer complex. Elongation of samples is produced when stress is applied. The predominant solid nature of all the samples have been exemplified as *G′* are greater than *G″* within the elongation range. Different evident trends are observed thereafter for LiTFSI–free and LiTFSI–based polymer electrolytes. For PMMA–PVC polymer blends solution, *G′* and *G″* values increase with percentage of strain insignificantly, up to 65%. However, above 65% strain, it is followed up by a minor decrease. It is suggestive of disruption of native interactive bonds within polymer matrix at high deformation. Nevertheless, upon addition of LiTFSI, these values decrease rapidly at high strain. This implies that the structures of LiTFSI–based polymer electrolytes can be broken down and deform easily when stress is applied on the samples. It might be ascribed to the plasticizing effect of LiTFSI. It softens the polymer backbone and then weakens the interactive bonds within the polymer matrix, causing a reduction in solid properties of polymer complex as the bonds can be broken readily. Upon inclusion of LiTFSI, at rest condition, both *G′* and *G″* values increase by more than an order of magnitude and hence this indicates the solid–like medium of samples. Hydrogen bonding might be an attributor to form the complexation between LiTFSI and polymer blends. As shown in Figure 4, the hydrogen atom of polymer blends is ready to react with oxygen atom of LiTFSI and contributes to the complexation. These hydrogen bonds initiate the entanglements within the polymer matrix and eventually lead to higher solid components in the samples.

As explained above, plasticizing effect of LiTFSI contributes to the reduction in solid behavior of the samples. The same phenomenon is observed in Figure 3. As expected, SPE 6 illustrates lower *G′* and *G″* values than SPE 3. *G′* and *G″* values decrease with increasing LiTFSI mass fraction. This effect reduces the crystalline region of the sample further. Amorphous is pre–dominant by weakening the interaction between the molecular bonding of polymer matrix. Consequently, the elastic properties of polymer electrolytes are decreased at high concentration of LiTFSI. However, by comparing SPE 6 and SPE 8, both values of *G′* and *G″* of SPE 8 are somewhat increased. It is primarily due to the precipitation of excessive salt in the polymer system. As a result, this salt agglomeration increases the solid nature of polymer blends systems. Among SPE 3, SPE 6 and SPE 8, SPE 6 illustrates the widest LVE range. It proposes the highest stability of SPE 6 and therefore it is difficult to be deformed.

### Oscillatory stress sweep

#### 1) First system

**Figure 5:**
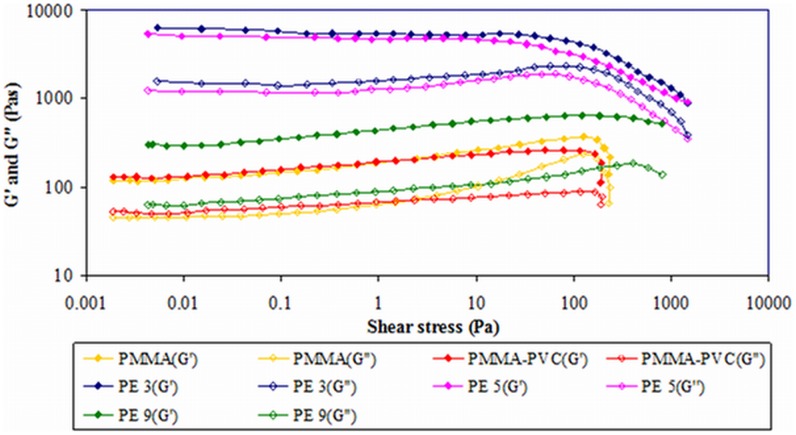
Oscillatory shear sweep of first system.

The plot of *G′* and *G″* versus shear stress of first system is depicted in Figure 5. Solid properties of all samples are verified by showing larger amounts of *G′* and *G″* within the range of shear stress. As expected, the flow point is absent and thus indicates the samples still remain in solid behavior. Pure PMMA shows an abrupt increase in *G″* at higher shear stress and thus asserts the increase in the liquid nature of sample when a large amount of stress is applied. Structural changes have been observed at high shear stress by demonstrating the decreases in *G′* and *G″*. The obtained result further verifies that the solid behavior of polymer electrolytes can be improved by adding PVC, as *G′* and *G″* of PMMA–PVC is higher than pristine PMMA solution. The result is in good agreement with the amplitude sweep. Pure PMMA and PMMA–PVC polymer solutions exhibit the lowest shear stress. This indicates that the lowest mechanical property of these polymer systems is due to the lesser forces is required to deform the samples. As can be seen, upon the incorporation of LiTFSI, the amount of shear stress required to start the deformation process is higher than pure PMMA and PMMA–PVC polymer solutions. It is due to more energy being required to break the hydrogen bonds of LiTFSI and polymer matrix as hydrogen bond is the strongest covalent bond.

Among PE 3, PE 5 and PE 9, PE 3 requires the highest shear stress, while PE 9 is the lowest. It can therefore be concluded that PE 3 is a stiff material as the transient covalent cross–linking between PMMA and PVC polymer backbones within this polymer matrix is difficult to be disrupted. However, stiffness of polymer systems decreases with PVC compositions. It is due to the aggregation of similar phase of PVC. As explained in section 3.1.1, this agglomeration causes the heterogeneous distribution within the polymer matrix and reduces the cross–linking effect. So, it might be divided into three regions, i.e., PMMA, PVC and combination of different ratio of PMMA–PVC polymer blends. Hence, the mechanical properties are reduced with the presence of the heterogeneous distributions.

#### 2) Second system

**Figure 6:**
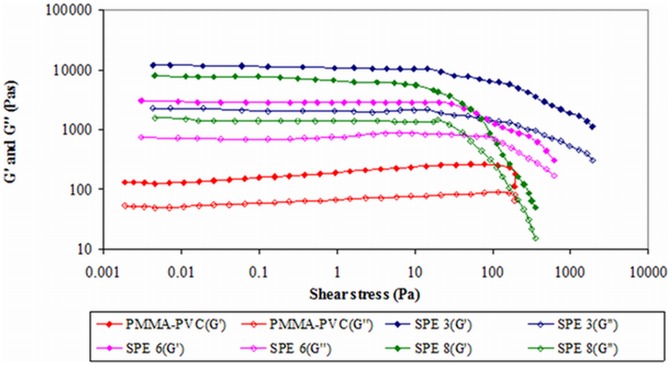
Oscillatory shear sweep of second system.

Figure 6 shows oscillatory shear sweep of second system. For all the samples, the values of *G′* are greater than *G″*. This indicates the domain solid properties in the polymer matrices. The crossover of *G′* and *G″* is absent within the range of shear stress. Again, this important feature indicates that there is no behavioral transition in the polymer electrolytes. Similarly, *G″* are increased upon dispersion of LiTFSI. It is evocative of the occurrence of complexation and further confirms the solid behavior of the samples in this oscillatory shear sweep. As explained in section 3.1.2, *G′* and *G″* values decrease with LiTFSI loadings, except for SPE 8. On the contrary, SPE 8 shows higher *G′* and *G″* values in comparison to SPE 6. These results are in good agreement with the amplitude sweep study of the second system. The first phenomenon is mainly due to the plasticizing effect, whereas the agglomeration of salt is for the latter trend. On the other hand, the sharp decrease at the end region is attributed to the destruction of the chemical bonding between the polymeric systems.

For the initial shear stress, PMMA–PVC illustrates the lowest value. In other words, the deformation of PMMA–PVC compound could be easily arisen by applying a small amount of force. Hence, it reveals the lowest mechanical stability. As explained above, the solid behavior of LiTFSI–based polymer electrolytes is increased. Therefore, the mechanical strength is also improved with impregnation of LiTFSI. It can further been proven in this study by increasing the amount of initial shear stress. It is expected that more energy is required to deform the LiTFSI–based polymer electrolytes due to the formation of coordination bonds between LiTFSI and polymer blends such as hydrogen bonds. However, SPE 6 depicts lower shear stress and thereby asserts the ease distortion of this sample. It might be associated with plasticizing effect of LiTFSI. In general, the plasticizing effect assists to destroy the dipole–dipole interactions, which in turn enhances segmental motion and flexibility of polymer matrix. Therefore, lesser energy is needed to break the bonds in the polymer complex. In contrast, the quantity of shear stress applied on SPE 8 is higher due to the salt agglomeration and this effect compensates the plasticizing effect. The salts have a tendency to form aggregation if the salts loadings are in excess. As a result, more stress is required to break the bonds among the aggregates, and thus the mechanical stability is enhanced.

### Oscillatory frequency sweep

#### 1) First system

**Figure 7:**
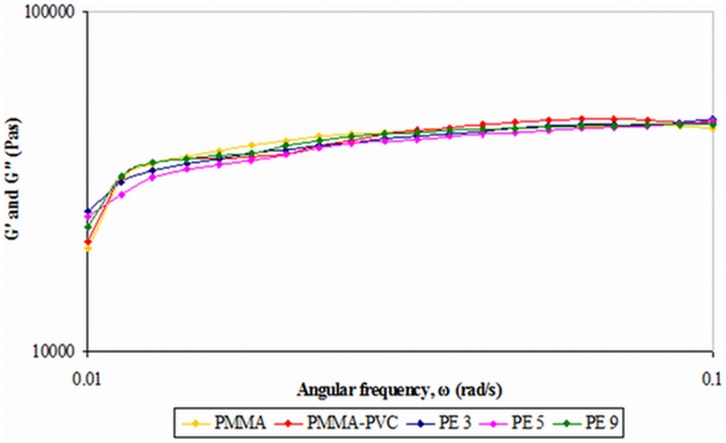
Frequency sweep of first system.

After amplitude sweep is investigated, the frequency sweep is also performed by using the proposed strain value. Figure 7 illustrates the frequency sweep of the first system. *G″* disappears in this frequency regime for all the samples. This denotes that the solid properties dominate in the sample. Two apparent regions are observed. First, an abrupt increase in *G′* occurs at low frequency whereas the frequency–independent relationship is attained at high frequency. In general, frequency is proportional to the reciprocal of time scale. At high frequency, all the samples show high *G′* values and this implies that the samples still maintain their strong gel and elastic characteristics in the short–term of deformation [2]. The high *G′* value at high frequency is due to the declined relaxation time. The polymer chains no longer slip past one another with this short relaxation time and the entanglements within the polymer matrix act like fixed network junctions. Thus, it increases the ability of this temporary polymer chain to store the temporarily imposed energy and leads to more elastic solid–like characteristic of polymer electrolytes [12]. On the other hand, *G′* decreases sharply with the frequency. It can be concluded that the elastic properties are significantly reduced and the long–term of structural stability becomes lesser. This might be due to the longer relaxation time and therefore the polymer chains can undergo the slippage process easily. Hence, it decreases the structural strength of macromolecules after a prolonged timescale.

Pure PMMA shows the lowest *G′* value at the lowest frequency of 0.01 rad s^−1^. It reveals that pure PMMA is the least stable candidate upon long–term deformation. However, the *G′* value is increased when PVC is embedded into the polymer. This is strongly connected to the cross–linking effect between PMMA and PVC. However, the solid–like nature increases further upon addition of LiTFSI. Among PE 3, PE 5 and PE 9, PE 3 shows the highest *G′* value and indicates the highest long–term structural stability. The *G′* value reduces with increasing PVC concentration. It is suggestive of the agglomeration of similar phase of PVC, which induces phase separation in the polymer matrices. This phase segregation reduces the formation of transient cross–linkage. The slippage of the intertwined polymer chains is more easily undergone as the cross–linking is lesser and this promotes the structural change within the polymer matrix. Therefore, the polymer electrolytes will not able to store the elastic energy, which is in accordance with lower value of *G′*.

#### 2) Second system

**Figure 8:**
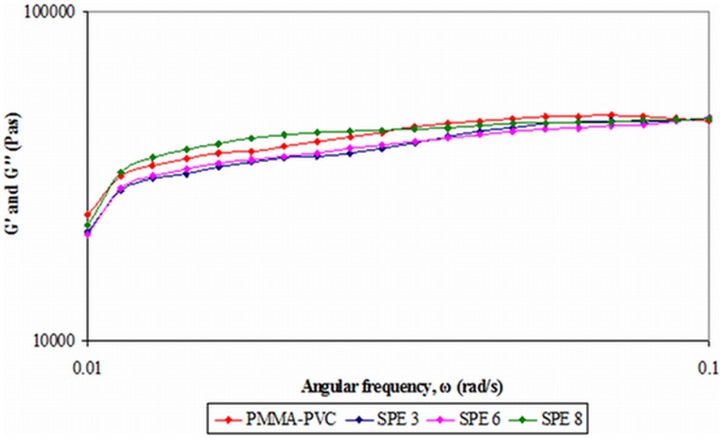
Frequency sweep of second system.

The frequency sweep of second system is shown in Figure 8. The disappearance of *G″* confirms once again the solid–like of all the samples. Again, same observations are obtained for the second system. Two visible regions are observed within the frequency range. *G′* exhibits frequency–dependence at low frequency, while an almost constant region is detected at high frequency. At high frequency, the frequency–independent regime with high *G′* value implied that there is no abrupt jump in elastic properties of sample when it undergoes the short–term deformation. It is principally endorsed to short relaxation time. The intertwined polymer chains are unable to slip past one another and act as fixed network junctions. Thus, it increases the ability of the polymer chain to store the imposed energy, leading to more elastic properties of the sample. In contrast, the structural strength has been declined when the deformation takes place for a prolonged time. The relaxation time becomes longer and the slippage of the entanglements of the polymeric chain is available. The ability of polymer chain to store the elastic energy is then reduced. As a result, the solid behavior becomes lesser at low frequency.

At the highest frequency of 0.1 rad s^−1^, *G′* values of all the samples are almost the same. In this stage, all the samples exhibit the same elastic energy and indicate that the addition of LiTFSI does not bring up any effect on the structural stability at high frequency. However, the *G′* values are varied as the frequency is decreased. This describes the different long–term structural stability of each sample. PMMA–PVC compound manifests higher structural stability by showing the least decrease in *G′*. The *G′* values are relatively decreased upon inclusion of LiTFSI. Comparing all LiTFSI–based polymer electrolytes, the increase in *G′* value is in this order: SPE 3 > SPE 8 > SPE 6. As can been seen, *G′* values decrease with increasing LiTFSI weight ratio, by comparing SPE 3 with SPE 6. Plasticizing effect of LiTFSI is the main contributor for the lowering in *G′* value. As explained in section 3.2.2, this effect aids in weakening the dipole–dipole interaction and breaks down the physical and chemical interactions within the polymer matrix more easily, promoting the mobility of polymer segments. Thus, the entanglements among the polymer matrix are more easily able to slip past one another. Therefore, it reduces the elastic solid–like properties of the polymer electrolyte. In conclusion, the solid–like behavior becomes lesser at low frequency. Surprisingly, *G′* value of SPE 8 is higher than SPE 6. It is suggestive of salt precipitation. The coordination bonds among the salt aggregates induce a difficulty on the slippage process. Therefore, the elastic properties are gradually increased.

### Viscosity

#### 1) First system

**Figure 9:**
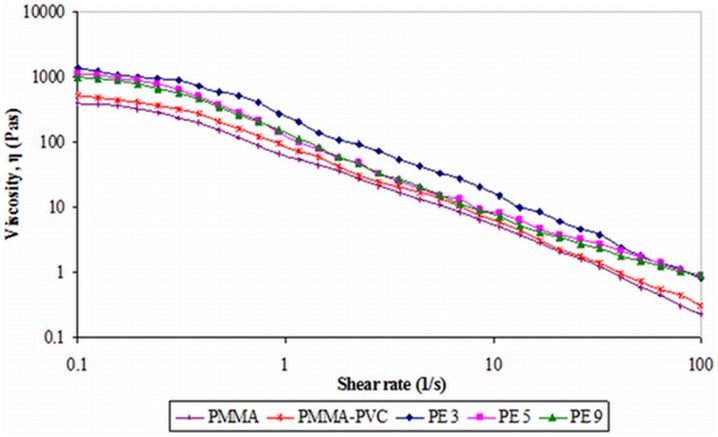
Typical viscosity curve of first system.

Figure 9 illustrates the typical plot of viscosity with respect to shear rate. As shown in Figure 9, the viscosities of all samples decrease with increasing shear rate, indicating the shear thinning properties of the samples [15]. This non–newtonian behavior indicates that the coordination bonds have been broken down as shear rate increases. In general, the viscosity of sample is high if the particular sample exhibits a solid–like behavior when small shear is applied. Pure PMMA behaves with lesser solid–like characteristic as proven in previous studies. So, it is expected that pure PMMA exhibits lower value of viscosity at low shear rate. By introducing PVC, the viscosity is increased. It is mainly ascribed to the formation of transient intercross–linking bond between PMMA and PVC. These cross–linkages reduce the polymeric chain mobility which is in accordance with an increase in viscosity [16].

On the contrary, the highest viscosity of PE 3 is further proven in this study as PE 3 shows the highest 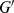 value. This enhancement of viscosity arises from the cross–linking between PMMA and PVC and the complexation between LiTFSI and polymer blends. However, at shear rate of 0.1 s^−1^, the viscosity of PE 5 and PE 9 are lower. It is indicative of aggregation of similar phase of PVC, which decreases cross–linking effect. For PMMA–PVC and PE 3, the shear thinning occurs as the cross-linkage is being disrupted by the shear. On the other hand, the non–newtonian behaviors of PE 5 and PE 9 are accredited to the combination of interruption of cross–linking and break down of weak physical bonds among the PVC aggregates. At highest shear rate of 100 s^−1^, the viscosity of PE 3, PE 5 and PE 9 are comparable. It means that the effect of PVC on viscosity is not so obvious at high rate of deformation. In other words, the viscosity is independent to PVC loadings at high shear rate.

#### 2) Second system

**Figure 10:**
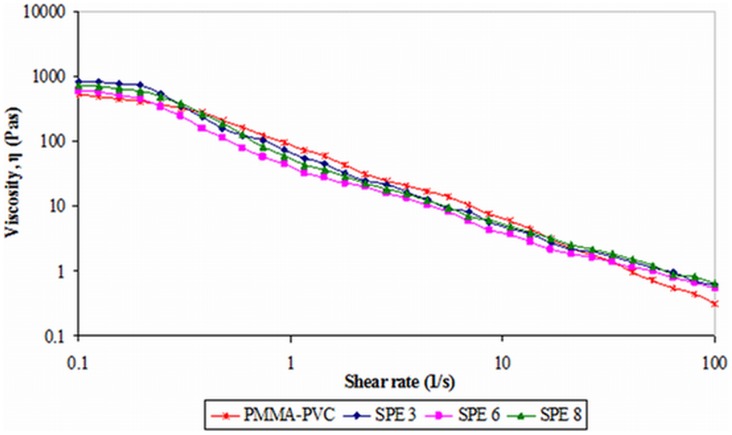
Typical viscosity curve of second system.

The viscosity of samples as a function of shear rate is depicted in Figure 10. At initial shear rate of 0.1 s^−1^, PMMA–PVC polymer blend shows the lowest viscosity, as shown in Figure 10. However, the viscosity is enhanced upon addition of LiTFSI due to the complexation between polymer blends and LiTFSI. As expected, the viscosity decreases with increasing the LiTFSI mass fraction, due to the plasticizing effect of LiTFSI. This effect softens the polymer backbone and hence increases the segmental mobility of polymer blends, favoring the disentanglements within the polymer matrix. As a result, it reduces the viscosity. However, the observed high viscosity in SPE 8 is against the theory. It is endorsed to the salt precipitation in the polymer system. Weak physical bonds are formed among the salt aggregates. It leads to a reduction of the movement of polymer chain and hence increases the viscosity by forming the network structure.

All samples exhibit downward shift in viscosity as shear rate increases implying the shear thinning behavior. This non–newtonian characteristic is due to the rupture of structure and bonding within the polymer matrix with increasing shear rate. It commonly denotes as disruption of inherent self–crosslinking between PMMA and PVC. For LiTFSI–based polymer electrolytes, the hydrogen bonding between LiTFSI and polymer blends has also been destroyed. The interactions between those excessive salt aggregates are interrupted for SPE 8 as well. The values of viscosity are almost the same at the highest shear rate of 100 s^−1^. This suggests that the addition of LiTFSI does not possess any contribution onto the viscosity when high shear is applied.

## Conclusion

In this work, two polymer electrolyte systems have been prepared. For first system, the ratio of PMMA to PVC is varied, whereas for second system, different mass fraction of LiTFSI is employed with a fixed ratio of polymer blends. In the amplitude sweep and oscillatory shear sweep studies, the values of *G′* are much higher than *G″*. This indicates the solid properties of polymer electrolytes. The absence of crossover of *G′* and *G″* indicates no behavioral transition in the samples. Pure PMMA shows the lowest mechanical properties with a smaller amount of *G′*. The mechanical properties of samples are improved upon addition of PVC and LiTFSI by showing higher *G′* values compared to amplitude sweep of pure PMMA. Elastic properties of polymer electrolytes decrease with frequency due to the longer relaxation time. This implies the lower structural strength of polymer electrolytes. In the oscillatory frequency sweep, *G″* is absent in the frequency regime and indicates the solid behavior of samples further. The viscosity of polymer electrolytes is reduced with increasing the shear rate and thus demonstrates the shear thinning properties. This is suggestive of disruption of cross–linkage, physical and chemical bonding in the polymer matrix.
